# Near-Peer-Teaching Revision Series During the COVID-19 Recovery Phase: An Experience From a UK Medical School

**DOI:** 10.7759/cureus.35775

**Published:** 2023-03-05

**Authors:** Pooja Devani, Arjuna Thakker, Nainal Shah

**Affiliations:** 1 Pediatrics, University Hospitals of Leicester National Health Service (NHS) Trust, Leicester, GBR; 2 Orthopedic Surgery, University Hospitals of Leicester National Health Service (NHS) Trust, Leicester, GBR; 3 Geriatrics, University Hospitals of Leicester National Health Service (NHS) Trust, Leicester, GBR

**Keywords:** education and training, medical school education, near-peer teaching, medical students, medical education

## Abstract

Background

The COVID-19 pandemic led to a significant disruption in clinical attachments for final-year medical students which can result in gaps in clinical knowledge and lower confidence levels. We developed a targeted near-peer-teaching (NPT) revision series to bridge this gap.

Method

A one-week virtual revision series was developed by the postgraduate doctors (PD and AT) and supervised by the final year written paper lead (NS) as per curriculum requirements. The series focused on eight key common clinical presentations. It was delivered via Leicester Medical School’s virtual platform by PD and AT a week before the finals. Multiple-choice surveys were sent out prior to the commencement of the series to gauge participation and baseline confidence. Surveys were also sent out before and after each session evaluating teaching, confidence levels, and improvement points.

Results

This NPT experience was the first comprehensive revision series during the COVID-19 recovery phase. Between 30 and 120 students attended each session. From the pre-series survey (n=63), almost all students indicated that their clinical attachments had been affected by the pandemic and that they (100%) would be interested in participating in the NPT series. From the post-session surveys, 93% of students reported that the session enhanced their confidence in recognizing and managing the clinical presentation, and 100% considered the quality of teaching to be good to excellent. From the post-series survey, a significant improvement was seen in confidence based on the Likert scale (from a combined 35% to 83%, pre, and post-series, respectively).

Conclusion

The series evaluation demonstrates that students found it a valuable experience and benefited from the social and cognitive congruence afforded by near-peer teachers. Furthermore, the results support the continued use and development of a virtual pre-exam revision series within the medical school curriculum as an adjunct to normal teaching.

## Introduction

The recent COVID-19 pandemic has not only brought about disruption to scheduled teaching in medical schools but has also changed the way students learn. Virtual learning environments (VLEs) are now a mainstay for delivering curriculum content and have advantages over traditional pedagogical teaching methods in flexibility and accessibility [1]. Furthermore, many VLEs can now facilitate active participation through the form of polls and chat boxes, encouraging dialogue and communication between students, teachers, and peers similar to face-to-face learning [2]. Some limitations with VLEs do exist and are mainly centered around technical infrastructure problems, lack of a social presence, and the academic maintenance of content [3].

Peer teaching (PT) and near-peer teaching (NPT) pedagogy has been studied formally within medical education literature, with their benefits seen in both undergraduate and postgraduate environments across healthcare disciplines [4]. Traditionally, PT can be defined as a group of individuals of similar educational levels, aiding one another’s learning. Near-peer teaching in concept is similar, however, those teaching are one or more academic years ahead of the person being tutored. The popularity of using both forms of teaching is growing as students find they can gain confidence in their learning within a controlled environment, a concept known as social congruence [5]. Benefits are also seen for those involved in teaching through the mechanism of knowledge consolidation as well as the development of core teaching skills. For clinicians, developing teaching pedagogy is desirable as set out in the General Medical Council’s Good Medical Practice [6]. For educational staff, PT and NPT help alleviate the pressure of the ‘faculty teaching burden’ in the face of growing curriculum and content demands.

As both pedagogical approaches in online learning and PT/NPT are valued, it was decided to create a focused online revision series. The series was designed for final-year medical students in 2022 at the University of Leicester, UK, and aimed to cover common clinical presentations aiding the students' preparations for their upcoming exams and also for their eventual transition to junior doctors. The past two years of clinical placements during the COVID-19 pandemic limited the patient-facing learning for this group of students. The series, which was not mandatory for students, was not used as a replacement for face-to-face tutorials but rather as an adjunct to scheduled teaching.

Two postgraduate doctors in conjunction with the final-year lead for written assessment developed the NPT content according to the final-year curriculum and learning outcomes. The series was then delivered by two postgraduate doctors (PD and AT). It was felt that final-year students would benefit from a near-peer leading the teaching rather than a middle-grade or consultant expert. This is because postgraduates are better placed to recall and relate to the understanding of the content required by the final year curriculum, exploiting the concept of cognitive congruence. This relatedness also helped facilitate an environment where concerns about understanding could be raised without the fear of being incorrect. This study was designed to evaluate the effectiveness of this NPT led by near-peer doctors.

## Materials and methods

Pre-series preparation

The series was delivered over one week, with each session and day designated to cover one or two common clinical presentations commonly encountered by foundation-year doctors (Table 1). The two postgraduate doctors created the content for each session with overall supervision from the final-year lead. Once the content and schedule had been finalized, a copy of the schedule was included in the weekly bulletin sent out via e-mail to all final-year medical students (December 2021). Regular reminders of the series were sent out leading up to the beginning of the allocated week of teaching (March 2022). One week before the start date of the series, a pre-series survey (see Appendix A) was sent out asking students to evaluate their confidence in managing common clinical scenarios (as per their curriculum learning outcomes) as well as to assess how their learning had been affected by the COVID-19 pandemic. Both were assessed using a numerical 5-point Likert scale to give quantitative value to qualitative data. Lastly, prospective participation in the upcoming series was gauged using the survey.

**Table 1:** Near-peer teaching (NPT) program topics GI: Gastrointestinal

Program Outline: Revision Series	
Proposed 5-day timetable teaching sessions that include medicine and surgery	
Day 1	Patient with chest pain (Chest Pain)
Day 1	Patient with edema (Acute/Chronic Heart Failure)
Day 2	Acute abdominal pain and GI bleeding (Surgical Emergencies)
Day 3	Patient with a high early warning score (Sepsis)
Day 3	Breathless patient (Shortness of Breath)
Day 4	Confused patient (Poisoning)
Day 4	Angry/agitated patient (Delirium)
Day 5	Patient with deranged sugars (Diabetic Emergencies)

Teaching

Before the start of each session, a link to a pre-session evaluation survey (see Appendix B) was displayed, which students were asked to fill out pre-session. The purpose of this was to determine the student’s confidence in recognizing and managing the topic to be covered prior to any teaching and to be able to quantify the change in confidence following the teaching session. Questions were asked using a typical Likert scale. Individual sessions were delivered virtually using the Blackboard Collaborate software (Blacboard Inc., Reston, VA, USA), which allowed live delivery and recording of the session whilst also facilitating discussion within the chat function between student and teacher. The two postgraduate doctors (PD & AT) alternated the teaching across the week and each session was overseen by the final-year lead (NS) virtually. Students were free to join and exit the session freely throughout the sessions. Each session within each day ranged from 45 to 60 minutes in length and was recorded so that it could be accessed later for revision purposes by students.

Reflection and evaluation

Following the completion of each session, a post-session evaluation survey (see Appendix C) validated by NS, was displayed for students to complete. The purpose of this survey was to query aspects of the NPT experience for students, such as the delivery and quality of content, relatedness to learning outcomes, and impact on clinical confidence. Similar to the pre-session survey, typical Likert scales were used. The post-session survey also provided critical feedback for the postgraduate doctors by identifying improvement points for the upcoming sessions.

Following the completion of the series, the post-series survey was sent out to all those who attended via the weekly bulletin. This final survey was used to assess whether the series improved students’ confidence in managing acute clinical presentations, whether the series helped students achieve their learning outcomes for the year, and whether the series made students feel more prepared for transitioning to a foundation doctor. This survey was also used to generate ideas concerning topics for future teaching.

## Results

Participants

Between 30 and 120 students attended each session. Numerous students participated in more than one session over more than one day. Official attendance was not taken for individual sessions or days. However, attendance was monitored by the supervising final-year lead. Days 1 and 5 were taught by one doctor (PD) whilst days 2, 3, and 4 were by the second doctor (AT).

Pre-series survey

**Figure 1:**
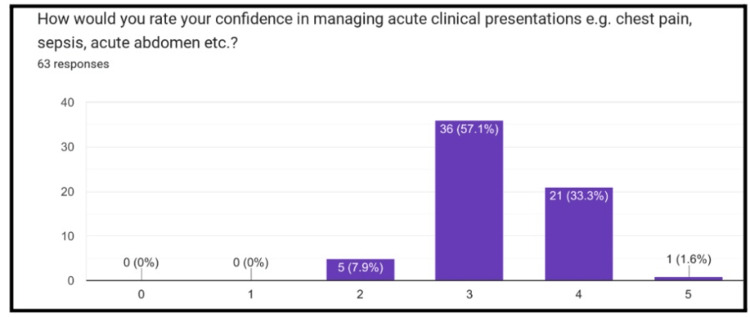
Likert scale assessing students’ confidence in managing acute presentations Numerical rating 0=not affected at all, 5=extremely affected

**Figure 2:**
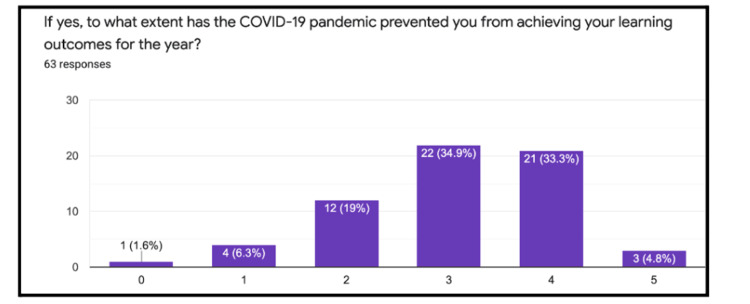
Likert scale assessing the impact of the COVID-19 pandemic on learning outcomes Numerical rating 0=not affected at all, 5=extremely affected

Sixty-three pre-series surveys were completed by the students. Of those who completed the survey, 57% reported a value of 3 on a 5-point Likert scale (0=not confident at all, 5=fully confident) when asked about their confidence in managing common clinical presentations (Figure 1). Furthermore, 98% of students reported that their clinical attachments had been affected by the COVID-19 pandemic. Following this, 98% of students reported that their ability to meet their learning outcomes for the year had been affected by the pandemic, with the majority of students choosing a 3 or 4 (0=not affected at all, 5=extremely affected) (Figure 2). Lastly, 100% of students who completed the survey felt that they would be interested in the proposed revision series.

Pre-session surveys

The number of students completing pre-session surveys varied between 10 to 40. The combined responses of ‘strongly agree and agree’ when students were asked if they feel confident in recognizing and managing the to-be-taught clinical presentations are shown in Table 2 and Table 3, respectively. A paired t-test was used to compare means between pre- and post-session responses. 

**Table 2:** Percentages showing students' perception of their confidence in recognizing each clinical presentation taught through the sessions, assessing their views before and after each session. Two-tailed p-value=0.0048 demonstrating statistical significance between responses

Recognising Clinical Presentation (Agree and/or Strongly Agree combined)	Chest Pain	Acute/Chronic Heart Failure	Surgical Emergencies	Sepsis	Shortness of Breath	Poisoning	Delirium	Diabetic Emergencies
Pre-session confidence (%)	86.8	66.7	76.2	87.5	78.3	53	63.7	66.7
Post-session confidence (%)	90.2	100	100	85.1	90.9	86.3	89.4	100

**Table 3:** Percentages showing students' perception of their confidence in managing each clinical presentation taught through the sessions, assessing their views before and after each session. Two-tailed p-value=0.0008 demonstrating statistical significance between responses

Managing Clinical Presentation (Agree and/or Strongly Agree combined)	Chest Pain	Acute/Chronic Heart Failure	Surgical Emergencies	Sepsis	Shortness of Breath	Poisoning	Delirium	Diabetic Emergencies
Pre-session confidence (%)	81.6	47.6	52.4	79.2	73.9	47.1	45.5	54.2
Post-session confidence (%)	91.8	100	100	88.9	97	95.4	88.9	96

Post-session surveys

The number of students completing the post-session surveys varied between 15 to 60. All those completing the surveys found the individual sessions relevant to their learning outcomes (100%). Their combined responses of ‘strongly agree and agree’ to whether the individual session increased the student’s confidence in recognizing and managing the taught clinical presentation. All the students considered the junior doctors to be knowledgeable in the topic they were delivering, answering either ‘strongly agree or agree’, and over 97% of students completing the post-session surveys would recommend the session to fellow students.

As part of the post-session survey, students were also asked to provide an overall rating for each session. The overall rating for each individual session is shown in Table 4, with the top two answers selected. The majority of students completing each post-session survey rated the session as ‘excellent’ or ‘very good’. No session was rated as ‘poor’.

**Table 4:** Individual session ratings using a qualitative scale from poor to excellent

Session (n=number of respondents)	Overall Rating	
Chest Pain (n=61)	Very good (41.7%)	Excellent (38.3%)
Acute/Chronic Heart failure (n=14)	Excellent (50%)	Very good (28.6%)
Surgical Emergencies (n=25)	Very good (50%)	Excellent (41.7%)
Sepsis (n=27)	Excellent (37%)	Very good (33.3%)
Shortness of Breath (n=33)	Excellent (50%)	Very good (34.4%)
Poisoning (n=22)	Excellent (54.5%)	Very good (18.2%)
Delirium (n=18)	Excellent (61.1%)	Very good (22.2%)
Diabetic Emergencies (n=26)	Excellent (42.3%)	Very good (38.5%)

For feedback on the sessions, qualitative themes and representative quotes are shown in Table 5. Some common trends that arose after each session that students found most beneficial included the interactivity of the sessions, the variety of clinical cases used, relevant and learning outcome-specific content, and the delivery of the session. Improvement themes centered around the speed of delivery, the use of a poll to answer questions, content not being up-to-date as per the latest guidelines, and the inclusion of more single best-answer questions.

**Table 5:** Qualitative themes and combined quotes from participating students when asked about positives and improvement points for the taught sessions SAQ: Self-assessment questionnaire, PE: Pulmonary embolism, NICE: National Institute for Health and Care Excellence

Themes	Positives	Improvements
Content	"Really great in-depth and relevant teaching'' | ''Focussed on preparing for finals and FY1''	"Quite a lot of content to cover in 1 hour, some things on slides weren’t fully explained'' | ''Clear images as CXR was blurry''
Delivery	"Pitched at a really good level, pace was fast enough to be able to get through the relevant topics but not so fast that I couldn’t keep up'' | ''The pace of the talk was good. Not too quick''	'Some SAQ-based questions would be beneficial'' | ''A little longer (time should be) spent explaining the management''
Interaction	"Interactive cases'' | ''Good interactions and to the point''	"Could try to use the poll function to ask questions as it gives more people a chance to answer rather than the same few people who answer really quickly in the chat'' | ''A few more interactive cases if possible''
Knowledge	"Was a very good speaker. Knowledgeable, ran to time, the perfect level for us'' | ''Knowledgeable''	"Management of PE is not up-to-date with new NICE guidelines'' | ''Answered questions but too much detail given beyond what our finals require''

Post-series survey

From the post-series survey (n=12), student’s overall confidence (Likert score of 3, 4, and 5 out of 5) in managing acute presentations improved from 92% (pre-series) to 100% (post-series), and a major improvement was seen in confidence based on the Likert scale with 4 and 5 combined (from 35% to 83% in pre and post-series. respectively). Furthermore, 92% of students (Likert scores 3,4, and 5) indicated that the series prepared them in making the transition to a junior doctor, (0=not helped at all, and 5=helped greatly). Other topics for future teaching included burns, pediatric emergencies, and trauma/orthopedics.

## Discussion

The ever-changing nature of the medical field necessitates the need to prepare our future clinicians. From advancing treatments to the exponential expansion of telehealth in our delivery of healthcare, there is no better time for medical education to also diversify to a virtual platform. With the pandemic over the last two years, this change has only become more necessary [7]. We describe the perceptions of our NPT revision series for final-year medical students at a UK medical school.

When assessing the pre-session survey responses, the survey demonstrated the impact of the COVID-19 pandemic on students’ clinical placements, with almost all students (98%) answering that their placements had been disrupted. This is unsurprising, given that during the height of the pandemic, medical schools responded by suspending clinical placements. Although this teaching series was delivered much after the pandemic’s peak, we expected residual adjustments to still be in place which would affect clinical placements. Given the high percentage demonstrated (98%), it is clear that these adjustments are very much in place and still impacting placements and having longer-term effects on students' education.

Furthermore, when assessing whether these impacts would affect students from meeting their learning outcomes, there was a clearly demonstrated skewed distribution towards the majority of students being ‘moderate to highly’ prevented when asked via a typical Likert scale. Surprisingly from this survey, despite the responses associated with the pandemic, students’ confidence in their ability to manage common clinical scenarios remained high. This suggests that despite the disruption of teaching, alternative methods of teaching that had been adopted by the medical school, such as VLEs, were working favorably.

From the results of the pre and post-session surveys, all presentations except for sepsis, student confidence in both recognizing and managing the clinical condition increased (p=0.0048 and p=0.0008, respectively) after the delivery of the session. The exception with sepsis could be attributed to the heavy teaching students often receive in pre-clinical and early clinical years on the topic (given its high prevalence and associated risk of mortality), making improvement difficult [8,9]. One hundred percent of the students also considered the doctors who delivered the sessions to be knowledgeable in the topic they were delivering. Additionally, over 97% of students completing the post-session surveys would recommend the session to fellow students. These results support the importance of cognitive congruence in the success of the NPT series. 

Evaluation of the series is not without its limitations. Firstly, there were two different postgraduate doctors delivering sessions. Despite both using the same virtual interface, teaching styles and therefore delivery of content were different. Furthermore, since feedback was collected and reviewed after every session, subsequent sessions were improved upon to benefit students, which could have resulted in detection bias when analyzing the responses. Secondly, whilst students may have attended the teaching, not all completed the feedback surveys. As we do not know this number, we cannot precisely account for the effects of non-responder bias. Lastly, we achieved a low response rate for the post-session survey. This could be improved upon by sending the survey straight after the series rather than waiting for students' exams to finish.

## Conclusions

With the advent of NPT forging a path to the hybrid model of teaching, we are now seeing both in-person and virtual teaching throughout higher education. This series was important in showing the benefit of NPT in the medical school setting, especially two years after the height of the pandemic, and to understand its utility. It would be important to analyze the improvement of confidence and knowledge for in-person teaching versus virtual to understand the true utility of virtual teaching.

## Appendices

Appendix A

Pre-Series Survey

1) How would you rate your confidence in managing acute clinical presentations e.g. chest pain, sepsis, acute abdomen, etc.?

Rate on  the Likert Scale: 0=not confident, 5=fully confident

2) How well do you feel your clinical placements have prepared you in making the transition to foundation year 1 doctor?

Rate on  the Likert Scale: 0=not prepared me at all, 5=fully prepared me

3) Have your clinical placements been impacted by the COVID-19 pandemic?

Answer yes/no

4) If yes, to what extent has the COVID-19 pandemic prevented you from achieving your learning outcomes for the year?

Rate on the Likert Scale: 0=not affected at all, 5=extremely affected

5) Would you be interested in attending further online webinars run by foundation year 2 and specialty trainee doctors to help consolidate your application of knowledge to clinical scenarios?

Answer yes/no

Appendix B

Pre-Session Survey

1) Which session is this for?

2) I feel confident in recognizing this clinical presentation

Choose: Strongly disagree, disagree, neither disagree nor agree, agree, strongly agree

3) I feel confident in managing this clinical presentation

Choose: Strongly disagree, disagree, neither disagree nor agree, agree, strongly agree

Appendix C

Post-Session Survey

1) Which session is this for?

2) The content of this webinar was relevant to my curriculum

Choose: Strongly disagree, disagree, neither disagree nor agree, agree, strongly agree

3) The aims and objectives of the webinar were clearly stated

Choose: Strongly disagree, disagree, neither disagree nor agree, agree, strongly agree

4) The presenter appeared knowledgeable about the content of the webinar

Choose: Strongly disagree, disagree, neither disagree nor agree, agree, strongly agree

5) The webinar has increased my confidence in recognizing this clinical presentation

Choose: Strongly disagree, disagree, neither disagree nor agree, agree, strongly agree

6) The webinar has increased my confidence in managing this clinical presentation as outlined in the year 5 curriculum

Choose: Strongly disagree, disagree, neither disagree nor agree, agree, strongly agree

7) One aspect of this webinar that you liked?

8) One aspect of this webinar that could be improved?

9) Overall, how would you rate this webinar?

Choose: Poor, fair, good, very good, excellent

10) I would recommend this webinar to a fellow student

Answer yes/no

## References

[1] AlQhtani A, AlSwedan N, Almulhim A (2021). Online versus classroom teaching for medical students during COVID-19: measuring effectiveness and satisfaction. BMC Med Educ.

[2] McKimm J, Jollie C, Cantillon P (2003). ABC of learning and teaching: web based learning. BMJ.

[3] Mukhtar K, Javed K, Arooj M, Sethi A (2020). Advantages, limitations and recommendations for online learning during COVID-19 pandemic era. Pak J Med Sci.

[4] Pintér Z, Kardos D, Varga P (2021). Effectivity of near-peer teaching in training of basic surgical skills—a randomized controlled trial. BMC Med Educ.

[5] Loda T, Erschens R, Loenneker H (2019). Cognitive and social congruence in peer-assisted learning—a scoping review. PLoS One.

[6] (2022). Ethical guidance - GMC. https://www.gmc-uk.org/ethical-guidance.

[7] Stoehr F, Müller L, Brady A (2021). How COVID-19 kick-started online learning in medical education-The DigiMed study. PLoS One.

[8] Ali K, Venkatasami N, Zahra D, Brookes Z, Kisielewska J (2022). Evaluation of sepsis teaching for medical and dental students at a British University. Eur J Dent Educ.

[9] Harrison DA, Welch CA, Eddleston JM (2006). The epidemiology of severe sepsis in England, Wales and Northern Ireland, 1996 to 2004: secondary analysis of a high quality clinical database, the ICNARC Case Mix Programme Database. Crit Care.

